# Lyophilized Preparation of Root Endophyte Fungus *Serendipita indica* Successfully Colonizes the Plant Host, *Cicer arietinum*

**DOI:** 10.1155/ijm/5609007

**Published:** 2026-07-18

**Authors:** Bindu Yadav, Pallavi Mourya, Rajeshwar Pratap Singh, Smriti Sri, Pratima Solanki, Atul Kumar Johri, Meenakshi Dua

**Affiliations:** ^1^ School of Environmental Sciences, Jawaharlal Nehru University, New Delhi, India, jnu.ac.in; ^2^ Special Centre for Nanoscience, Jawaharlal Nehru University, New Delhi, India, jnu.ac.in; ^3^ School of Life Sciences, Jawaharlal Nehru University, New Delhi, India, jnu.ac.in

**Keywords:** carbon dots, *Cicer arietinum*, lyophilization, SDGs, *Serendipita indica*

## Abstract

Root endophyte fungus *Serendipita indica* can be axenically cultivated, is easily obtained in pure cultures in the laboratory, and therefore can be developed as a biofertilizer for bioaugmentation. In this study, an effort towards sustainable organic agriculture, we have made two completely eco‐friendly, biogenic, and biocompatible lyophilized formulations of *S. indica*: one without carbon dots and the other with carbon dots. Scanning electron microscopy (SEM) observations, viability assays, and colonization efficiency of both the formulations revealed that lyophilization does not in any way alter either the morphology, growth, or colonization ability of the endophyte. The formulations were also tested for impact on growth of *Cicer arietinum* plants in experimental setup. The plants were analyzed for changes in dry weight, shoot length, root length, and branch numbers. The dry weight increased by a maximum of 1.9‐fold, average shoot length by 1.4‐fold, average root length by 1.7‐fold, and number of branches by 1.4‐fold, when compared with plants grown without any *S. indica*. These increases were found to be statistically significant. We identify this work as a significant step towards optimizations and production of this formulation on a larger scale. We also perceive this attempt as commitment towards United Nations SDGs 2, 3, and 13.

## 1. Introduction

*Serendipita indica* is a root endophyte that colonizes both monocots and dicots. The fungus was isolated from the rhizosphere of xerophytes in Rajasthan′s Thar Desert in the western part of India [1]. It helps the colonized plants alleviate a variety of abiotic and biotic stresses; it has time and again been advocated variously as a plant probiotic, natural plant growth promoter, immune modulator, metabolic regulator, biofertilizer, phytoremediator, antioxidant enhancer, bioherbicide, bioinsecticide, and biopesticide. It confers resistance against abiotic and biotic stresses, at the same time enhancing plant productivity [2–7]. Its role as bioprotector conferring systemic resistance to the plants against several toxins, heavy metals, and pathogens is well documented [8–11]. The 25‐Mb genome of *S. indica* has been sequenced and annotated [12]. *S. indica* can be axenically cultivated and is easily obtained in a pure culture in the laboratory. Given so many credits to its being, it is possible that laboratory‐grown *S. indica* can be developed into a suitable formulation to be used as a biofertilizer for field applications.

Lyophilization preserves the vitality as well as stability of microorganisms over long periods of time by reducing their moisture content through vacuum freeze‐drying [13]. Lyophilization increases the shelf life of the microbial samples and makes them convenient for transport. A lyophilized formulation of *S. indica* fungal biomass can ease its mixing in the farm soil, but the real challenge is survival of the lyophilized preparation in soil till the endophyte can find a suitable host.

Agricultural nanotechnology is emerging to be a promising technique; yet, concerns about fate, transport, bioavailability, and toxicity of nanoparticles, as well as inadequacy of the regulatory framework, limit the agricultural sector′s willingness to adopt such technology [14, 15]. However, the use of nanotechnology, including biogenic synthesis of carbon‐based nanomaterial including carbon dots (CDs), multiwalled carbon nanotubes, and biopolymer‐based fertilizers, can be suitable alternatives to direct use of chemical fertilizers [16–18]. As arbuscular mycorrhizal fungi (AMF) have already been used as biofertilizers [19], it makes good sense to use fungal microbes containing CDs in preparing bioinoculant formulations for field application as a step towards sustainable agriculture. CDs are proven biocompatible and biodegradable, highly stable particles for green synthesis [20]. These can be easily taken up by biological cell membranes and cause negligible damage to cell integrity [21]. Additionally, CDs exhibit excitation‐independent photoluminescence (PL) and are highly autofluorescent even when exposed to low UV light, thereby making them ideal in application with bioimaging and tracking [22]. Therefore, in the present study, we propose preparing biocompatible and eco‐friendly lyophilized formulations of *S. indica* for field applications. In this study, we describe two such formulations: first, lyophilized fungal biomass not containing CDs (henceforth referred to as *untreated* fungal biomass) and, second, lyophilized fungal biomass containing CDs (henceforth referred to as *treated* fungal biomass). The work done towards designing and testing these formulations includes synthesis of nontoxic, biocompatible CDs, internalization of the CDs in *S. indica* fungal biomass, lyophilization of the *untreated* and *treated* fungal biomass, viability testing of the *untreated* and *treated* fungal biomass in liquid broth as well as on agar plates, and interaction of lyophilized preparations with the plant host *Cicer arietinum* (chickpea) to check colonization efficiency as well as impact on biomass of the plants.

## 2. Results

### 2.1. Characterization of CDs

**Figure 1:**
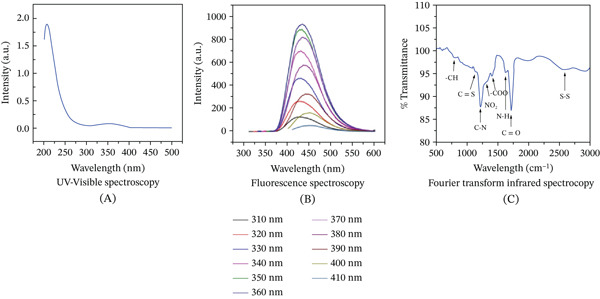
Characterization of carbon dots. (a) UV‐Visible spectroscopy. (b) Fluorescence spectroscopy. (c) Fourier‐transform infrared spectroscopy.

The UV absorption peaks at 360 nm (Figure 1a) because of the n–*π* ∗ transition of C=O bonds. This n–*π* ∗ is responsible for PL. The PL emission spectra of CDs were recorded at different wavelengths from 310 to 410 nm (Figure 1b). The maximum emission of CDs was centered at 443 nm when excited at 360 nm. The functional groups of CDs were analyzed using Fourier‐transform infrared spectroscopy (FTIR) spectroscopy. IR peak at 3340 cm^−1^ was attributed to the stretching vibration of NH_2_ of L‐cysteine. The bending vibration of N–H was observed at 1652, 1570, 1558, and 1542 cm^−1^. Strong absorption IR peaks observed at 1716, 1731, and 1635 cm^−1^ were assigned to the C=O stretch. Absorption bands at 1223 cm^−1^ were because of C–O–C stretch. The presence of sulfur in CDs was confirmed by the absorption peak at 2600 and 635 cm^−1^. The FTIR spectra confirmed that the CDs contained the aromatic ring of carbon and carboxylic acid, primarily amine and sulfur as the functional groups corresponding to CA and L‐cysteine (Figure 1c). The zwitterionic nature of CDs was confirmed from the zeta potential studies of CDs. The overall charge of CDs was found to be 89 mV.

**Figure 2:**
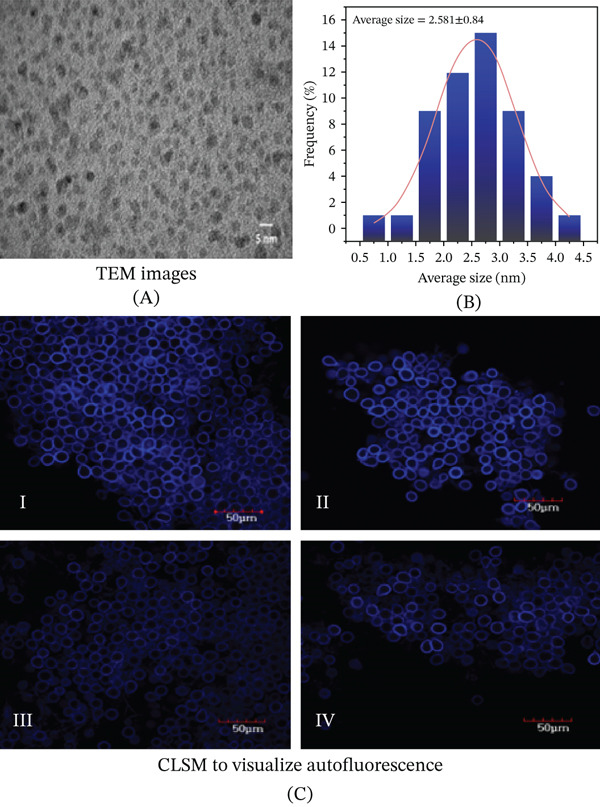
(A) TEM imaging of CDs. (B) Size distribution of CDs. (C) Autofluorescence in spores as visualized by CLSM after treating *S. indica* with varying concentrations of (I) 200 *μ*g/mL CDs for 2 h, (II) 400 *μ*g/mL CDs for 2 h, (III) 600 *μ*g/mL CDs for 2 h, and (IV) 800 *μ*g/mL CDs for 2 h.

TEM images showed that CDs are uniformly distributed over the surface (Figure 2a). The particles showed a narrow size distribution varying from 1.5 to 4 nm. The average particle size was found to be 2.5 ± 0.8 nm (Figure 2b). CDs exhibited well‐resolved lattice fringes with *d*‐spacing of 0.206 nm. This lattice spacing, being the characteristic of graphitic carbon (100) facet in CDs, showed that CDs have crystallinity. However, we also observed that not all CDs were crystalline in nature. The selected area electron diffraction (SAED) pattern showed that most of the CDs were amorphous in nature.

### 2.2. Internalization of CDs by *S. indica*

The internalization of CDs by *S. indica* was confirmed by autofluorescence of CDs. We found uptake of CDs in *S. indica* was time‐ and concentration‐dependent. The maximum visible fluorescence was observed in fungal biomass *treated* with 400 *μ*g/mL CDs for 2 h (Figure 2c). We found that any increase in the concentration of CDs beyond 400 *μ*g/mL or time beyond 2 h resulted in an actual decrease in autofluorescence. This could be because longer incubations or higher concentrations of the CDs likely result in a reverse flow from fungal cells back into the suspension.

**Figure 3:**
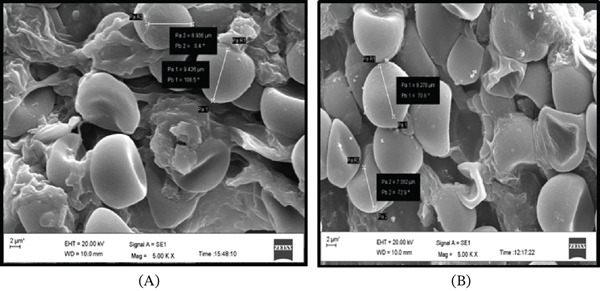
SEM of (A) lyophilized *S. indica* (*untreated* fungal biomass) and (B) lyophilized *S. indica* with 400 *μ*g/mL CDs for 2 h (*treated* fungal biomass).

Our scanning electron microscopy (SEM) data show that CDs did not have any adverse effects on *S. indica* morphology because in both the *treated* and *untreated* fungal biomass, the pear‐shaped structure of fungal spores was found to be intact (Figure 3A,B).

### 2.3. Lyophilization of *Treated* and *Untreated* Fungal Biomass

**Figure 4:**
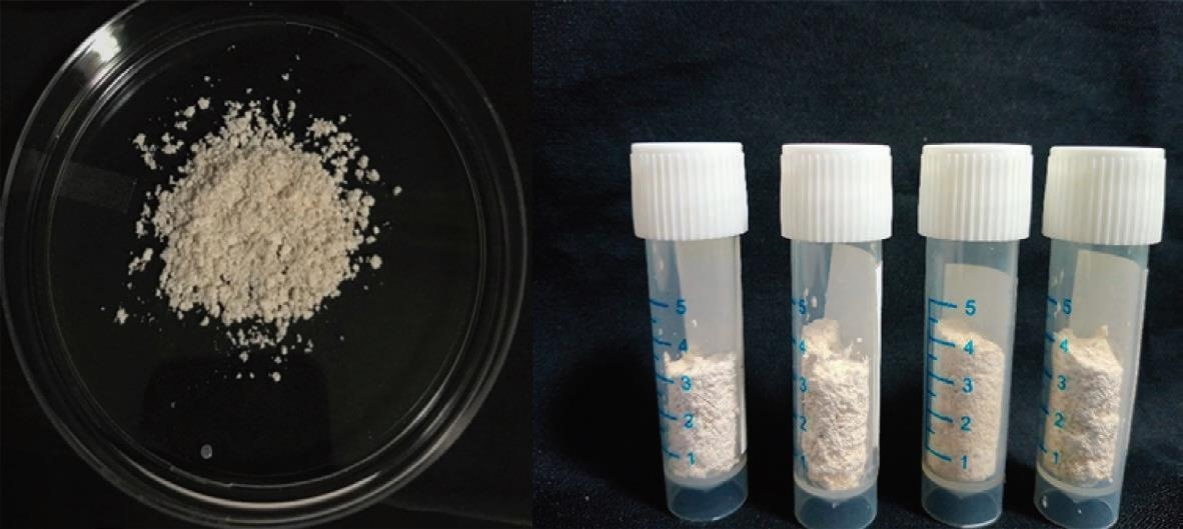
Lyophilization of *S. indica*. Pale yellow powder in multiple aliquots.

**Figure 5:**
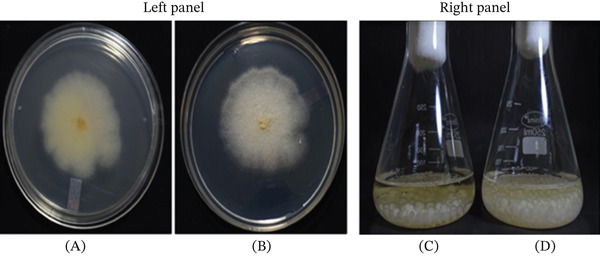
Viability assay of lyophilized *S. indica* under laboratory condition after 2 weeks of lyophilization. Left panel: (A) Culture growing from lyophilized *S. indica* on KF medium agar plates. (B) Culture growing from lyophilized *S. indica* treated with 400 *μ*g/mL CDs for 2 h on KF medium agar plates. Right panel: (C) Lyophilized *S. indica* growing in KF broth. (D) Lyophilized *S. indica* treated with 400 *μ*g/mL CDs for 2 h, growing in KF broth.

The *treated* as well as *untreated* fungal biomass was lyophilized to a fine pale‐yellow powder under sterile conditions (Figure 4). Multiple batches were run to generate aliquots of the powder. This lyophilized preparation was checked for viability on agar plates as well as in broth at different time points: immediately, 1 week, 2 weeks, and 1 month after lyophilization. All the lyophilized preparations grew to maturity in 21 days just like the routine batch cultures of *S. indica* (Figure 5A–D).

### 2.4. Lyophilized *S. indica* Successfully Colonized Plants

**Figure 6:**
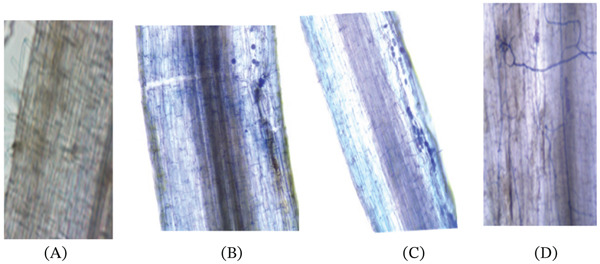
Colonization analysis in the roots of chickpea plants. (A) Plants grown without any *S. indica*, (B) plants colonized with fresh *S. indica*, (C) lyophilized *S. indica* (*untreated* fungal biomass), and (D) lyophilized *S. indica* with 400 *μ*g/mL CDs for 2 h (*treated* fungal biomass).

We observed that although roots that were not exposed to *S. indica* did not show, as expected, the presence of spores (Figure 6A), the colonization percentage in plant roots exposed to fresh *S. indica* was found to be ~90% (positive control) (Figure 6B). Most encouragingly, the colonization efficiency of lyophilized *S. indica* (*untreated*) in plant roots was found to be 70% in 40 days (Figure 6C). This set of plants did not reach any higher colonization percentages possibly because of the time lag lyophilized *S. indica* may have taken to grow from desiccated state into vegetative stage before further entering into sporulation. It was also observed that many of the root sections where *treated* biomass was used showed comparable colonization, that is, 75% with that of *untreated S. indica* (Figure 6D). The reason could be the same as the premise taken for this study that CDs reduce the dependence of fungus on plant host for fixed C, which in turn either delays its interaction or prolongs its vegetative stage in the host. Nonetheless, further in‐depth studies at a molecular level are needed to understand the reason behind extensive hyphal growth before sporulation inside the host roots, when fungus is treated with CDs.

### 2.5. Lyophilized *S. indica* Enhances Growth of the Colonized Plants

**Figure 7:**
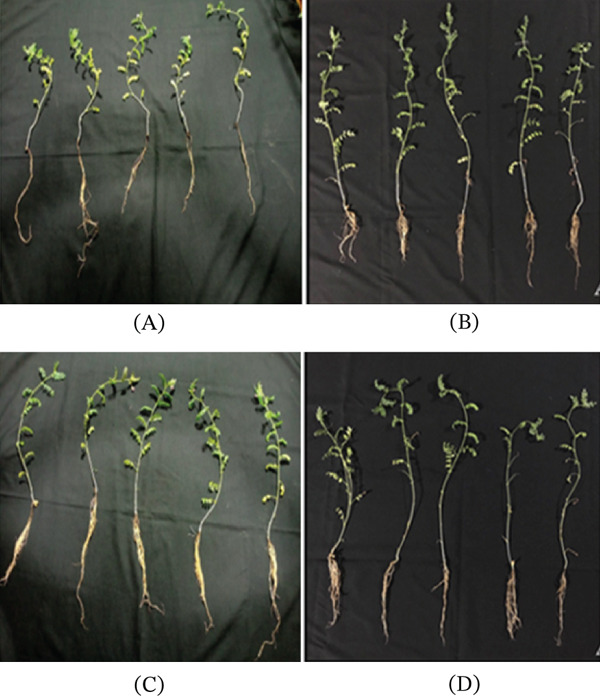
Growth and biomass analyses. (A) Plants grown without any *S. indica*, (B) plants grown only with fresh cultures of *S. indica* mixed with soil at a final concentration of 0.1% (*w*/*w*), (C) plants grown with lyophilized *untreated* fungal biomass mixed with soil at a final concentration of 0.1%, and (D) plants grown with lyophilized *treated* fungal biomass mixed with soil at a final concentration of 0.1% (*w*/*w*).

We found that plants colonized with the fresh cultures of *S. indica*, lyophilized *untreated S. indica*, and lyophilized *treated S. indica* showed morphologically better growth and biomass in comparison with the noncolonized plants (control) (Figure 7A–D). Growth parameters were analyzed for dry weight, shoot length, root length, and branch numbers.

**Figure 8:**
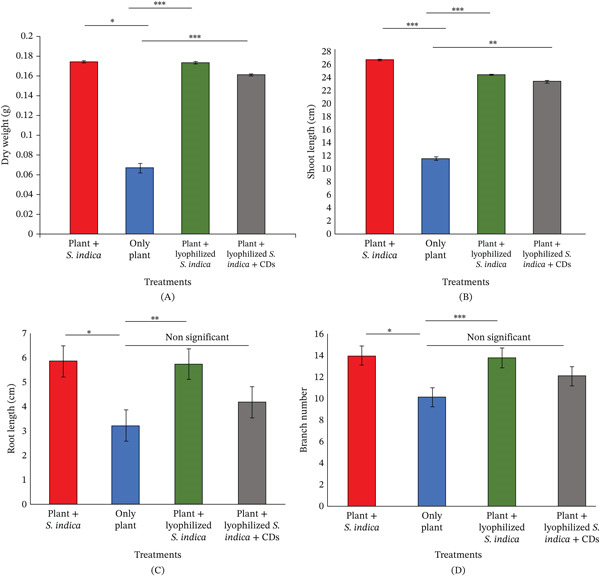
Plant growth and development analyses. (A) Dry weight, (B) shoot length, (C) root length, and (D) number of branches. Data are shown as mean ± SEM (*n* = 3 biological replicates and three technical replicates). The statistically significant differences are marked with asterisks  ^∗^ *p* < 0.05,  ^∗∗^ *p* < 0.01,  ^∗∗∗^ *p* < 0.001. Unpaired *t*‐test was conducted using Prism Software Version 8 (GraphPad).

The dry weight of plants grown with lyophilized *untreated* fungal biomass as well as plants grown with fresh fungal cultures showed an equal increase of 2.5‐fold when compared with plants grown without any *S. indica* (control). The dry weight of plants grown with lyophilized *treated* fungal biomass showed a 2.3‐fold increase when compared with plants grown without any *S. indica* (control). All these differences were found to be statistically significant (*p* < 0.001) (Figure 8A).

The average shoot length of plants grown with lyophilized *untreated* fungal biomass and those grown with fresh fungal culture increased by 2.1‐ and 2.3‐fold, respectively, when compared with plants grown without any *S. indica* (control). These differences were found to be statistically significant (*p* < 0.001). The shoot length of those grown with lyophilized *treated* fungal biomass showed a two‐fold increase over plants grown without any *S. indica* (control). This difference was also found to be statistically significant (*p* < 0.01) (Figure 8B).

The average root length of plants grown with lyophilized *untreated* fungal biomass and those grown with fresh fungal culture increased by 1.7‐ and 1.9‐fold, respectively, when compared with plants grown without any *S. indica* (control). These differences were found to be statistically significant (*p* < 0.01 and *p* < 0.001, respectively). However, the root length of those grown with lyophilized *treated* fungal biomass did not show any statistically significant increase (Figure 8C).

The number of branches in plants grown with lyophilized *untreated* fungal biomass and those grown with fresh fungal culture increased equally by 1.4‐fold when compared with plants grown without any *S. indica* (control). These differences were found to be statistically significant (*p* < 0.001). However, the number of branches in plants grown with lyophilized *treated* fungal did not show any statistically significant increase (Figure 8D).

## 3. Discussion

It has been established that excessive use of chemical fertilizers and pesticides results in significant changes in soil texture and fertility, which impact crop yield all over the world [23]. In order to develop high crop yield with increased nutritious value, the use of biofertilizers has been suggested for sustainable agriculture and economic benefits. In this regard, plant‐probiotic endophytic fungus *S. indica* has been found to colonize a wide range of cereal as well as medicinal plants and help them against various abiotic and biotic stresses, hence promoting plant growth and development [2, 8, 10, 11, 24]. Recently, *S. indica* was used as a straw‐based fertilizer and has been suggested as a practical approach for field application of *S. indica*–based fertilizers [25, 26]. In addition, it has been found that along with humic acid, *S. indica* can help tomato plants against metal toxicity and improve fruit quality [27, 28]. Recently, the effects of nano Fe‐oxide and *S. indica* on degradation of petroleum hydrocarbons in soil and thereby reducing arsenic concentration in barley plants have been shown [29]. Application of inorganic carrier‐based formulations of fluorescent pseudomonads and *S. indica* on tomato plants has been tested [30], wherein culture broths of pseudomonads R62, R81, and *S. indica* were successfully used for development of talcum‐ and vermiculite‐based bioinoculant formulations. Further, talcum‐based bioinoculant formulations were found to be significantly better than the vermiculite‐based formulations. Though these studies suggest that *S. indica* can be used as a biofertilizer, a closer look at these formulations reveals that these studies have not described the stability, viability, and transportation of the formulation.

*S. indica* has great potential to be used as a biofertilizer in increasing biomass and productivity of crops when compared with other mycorrhiza including the AMFs [23]. Though found natively in soils, *S. indica* faces all the usual challenges presented by a complex ecosystem as soil, towards growth and colonization of its host. Therefore, to be able to put it to maximally beneficial use in agriculture, we suggest bioaugmentation of this endophyte in agricultural farmlands. In our opinion, field application requires a suitable formulation that is eco‐friendly, sustainable, easy to add in the soil, can increase the shelf life of the fungus to give it sustained longevity in the absence of a host, and can also be scaled up for commercial use. For the first time, we have successfully attempted to make such a working formulation by lyophilizing the fungal biomass harvested from laboratory‐grown pure cultures of *S. indica*. Further, to strengthen its stability, viability, and efficiency after addition in the soil, we have integrated nano‐sized, photostable, nontoxic, and biocompatible CDs inside the fungal biomass before lyophilization. Indeed, as our results show, lyophilized *S. indica* is revivable in laboratory cultures and not only thrives just as well as the routine fresh batches, there is also no change in either the growth rate or morphology of the spores. Moreover, like fresh cultures, the lyophilized formulation is also able to colonize the chickpea plants efficiently and is comparable to it in its positive impact on plant growth and biomass. Further, it is important to note that even though lyophilization of the fungal biomass with CDs inside did show good revival and resulted in significant increase in dry weight and shoot length of the colonized plants, the CDs did not confer any additional advantage to fungal revival and colonization. Simple lyophilized preparation without pretreatment with CDs is sufficient to retain colonization potential and impact on plant dry weight, shoot length, root length, and branch numbers. Previously, use of carbon nanoparticles, curcumin‐based carbon nanodots (Cur‐CND), and glycine‐based carbon nanodots (Gly‐CND) along with mycorrhizal fungi have been found to improve plant growth by increasing their nutrient content and photosynthetic activity [31–33]. Our study not only confirms the assertions of the aforementioned reports but also moves a step further in demonstrating that simple lyophilization, without expensive nanomaterial pretreatments, preserves the bioefficacy and functional colonization capacity of *S. indica*. However, before making any conclusions, it will be worthwhile to expand this study over a longer period of plant growth to also include the reproductive phase and fruiting. In a parallel, yet unpublished study done in our laboratory, it has been shown that addition of axenically grown *S. indica* does not alter the microbial diversity of the soil, therefore giving a more promising prospect towards its bioformulation. This preliminary work, to the best of our knowledge also a first for lyophilization of *S. indica*, certainly entails optimization of shelf life, survival, colonization efficiency, and larger in situ experimental cohorts before scaling up the process for bioaugmentation on commercial scale. The larger aim is utilizing the potential of *S. indica* in sustainable organic agriculture to achieve food security, enhanced productivity, and health of food crops in the face of climate change, as also realized in United Nations SDGs 2, 3, and 13.

## 4. Material and Methods

### 4.1. Fungal Culture

*S. indica* culture was obtained from Prof. Ajit Varma, Amity University, Noida, UP, India^1^. *S. indica* cultures were routinely maintained both in KF culture medium broth (20 g L^−1^ glucose, 2 g L^−1^ peptone, 1 g L^−1^ yeast extract, 1 g L^−1^ casamino acid, 520 mg L^−1^ KCl, 520 mg L^−1^ MgSO_4_7H_2_O, 1.52 g L^−1^ KH_2_PO_4_, 55 mg L^−1^ ZnSO_4_7H_2_O, 27 mg L^−1^ H_3_BO_3_, 12 mg L^−1^ MnCl_2_4H_2_O, 4 mg L^−1^ CoCl_2_6H_2_O, 4 mg L^−1^ CuSO_4_5H_2_O, 16.2 mg L^−1^ CaCl_2_, 16.2 mg L^−1^ FeCl_3_, 0.5 mg L^−1^ biotin, 0.5 mg L^−1^ nicotinamide, 1 mg L^−1^ pyridoxal phosphate, 1 mg L^−1^ aminobenzoic acid, 2.5 mg L^−1^ riboflavin, and 2% agar; pH 6 ± 0.5) and on agar plates [33]. *S. indica* was grown in liquid KF medium in a 250‐mL culture flasks under constant shaking at 110 rpm and at 30°C for 7–9 days in a metabolic shaker (Orbitek shaker, India).

### 4.2. Synthesis and Characterization of CDs

To synthesize CDs, citric acid (0.52 M) and 20.8‐mM L‐cysteine were dissolved in 10‐mL ultrapure water as described [22, 34]. The clear solution was placed in a microwave for 3 min at 700 W. After allowing the yellow precipitate to cool at room temperature, 5‐mL ultrapure water was added to dissolve it, yielding a yellow color solution with the desired CDs. To remove impurities, formulation was dialyzed against pure water for 3 days using a dialysis membrane (Spectra/Por MWCO 1000 Da). Finally, a solution containing CDs was oven‐dried at 80°C, yielding 0.157‐g CDs. From this, 1‐mg CDs were dispersed in 1‐mL ultrapure water before being used. The UV‐Vis and fluorescence spectra of CDs were measured using a T90+ UV/vis spectrometer (PG Instruments Ltd, United Kingdom). The fluorescence behavior of CDs was examined using a Nikon real‐time confocal laser scanning microscope (A1R). High‐resolution transmission electron microscopy (HRTEM) (JEOL JEM‐2200 FS, Japan) was used to characterize the size and crystallinity of CDs. Because the functional group in the CDs correspond to citric acid and L‐cysteine, the FTIR confirmed that the CDs include the aromatic ring of carbon and carboxylic acid, mainly amine and sulfur.

### 4.3. Internalization of CDs by *S. indica*

*S. indica* being a root endophyte fungus is dependent on its plant host for fixed carbon, which implies that without a host the endophyte will either have limited survival or may not survive altogether. Therefore, laboratory‐synthesized CDs, as a source of fixed carbon, were internalized in the lyophilized fungal biomass in a bid to ensure its survival in the field till the time it can find and colonize the plant host roots. *S. indica* fungal biomass was harvested from culture agar plates by flooding the plates with liquid KF broth and carefully scraping the fungus with a sterile glass spreader in a centrifuge tube. Small hyphal fragments were removed by centrifugation. The supernatant was discarded, and the pellet obtained (*untreated* fungal biomass) was diluted with sterile distilled water. The number of spores was quantified under a light microscope using a hemacytometer (Neubauer Scientific, Pennsylvania, United States). Concentration‐ and time‐dependent study was performed to check the uptake of CDs. To investigate the uptake of CDs by *S. indica* at various concentrations, varying concentrations of CDs (1000, 800, 400, 200, 100, 50, and 25 *μ*g/mL) were added to the fungal biomass in separate wells and incubated for various time periods (0, 1, 2, 4, and 6 h) (*treated* fungal biomass). Autofluorescence in spores was visualized by confocal laser scanning microscope (Olympus FluoView FV1000) at 40x excited by 405‐nm laser. Further, both *treated* and *untreated* samples were viewed under SEM (EVO 18 special edition, ZEISS) to check for any morphological changes post internalization of CDs. To do SEM, samples were rinsed with PBS buffer, placed in fixative (2.5% glutaraldehyde) for 4 h, washed again with PBS buffer, and stored overnight at 4°C. Further, samples were dehydrated in an ascending series of 10% increments from 50% to 100% ethanol for 20 min each before being kept for drying. Only fungal biomass served as positive control.

A viability check was done to test the viability of fungal biomass after internalization of CDs. For this purpose, variously *treated* fungal biomass defined by different concentrations and time was grown on separate KF agar plates and liquid broth at 30^°^C ± 2^°^C for 10 days. Fungal biomass incubated only with distilled water for 2 h was used as control.

### 4.4. Lyophilization of *S. indica*

For this purpose, 100‐mL broth culture of both *untreated* and *treated* fungal biomass was separately sieved, washed using sterile distilled water, and transferred to sterile lyophilization tubes. The tubes were kept frozen in liquid nitrogen for 2 min. Tubes were uncapped and covered with sterile aluminum foil and loaded on the lyophilizer (Operon FDB 5503) at temperature and vacuum of −57°C and 10^−3^ Torr, respectively, for 24 h. The lyophilized powder was stored in batches of 10 mg in sterile capped vials at 4°C. To test the viability of lyophilized preparations, 10 mg each of *untreated* and *treated* lyophilized fungal biomass was inoculated separately on KF agar plates (incubated at 30°C in an incubator) and in KF broth incubated at 30°C, 111 rpm in a metabolic shaker (Orbitek shaker, India).

### 4.5. Interaction of *S*. *indica* With Plant

To find out if lyophilization or the CDs have any effect on the ability of *S. indica* to interact with the host plant, four sets of pots were prepared: (1) Plants were grown without any *S. indica*, (2) plants were grown only with fresh cultures of *S. indica* mixed with soil at a final concentration of 0.1% (*w*/*w*), (3) plants were grown with lyophilized *untreated* fungal biomass mixed with soil at a final concentration of 0.1% (*w*/*w*), and (4) plants were grown with lyophilized *treated* fungal biomass mixed with soil at a final concentration of 0.1% (*w*/*w*).

Chickpea plant (*C*. *arietinum*) seeds were purchased from Indian Agriculture Research Institute, Pusa, New Delhi. To grow chickpea plants, seeds were surface sterilized. To do this, approximately 50 seeds were soaked in 100‐mL sterile distilled water for 1 h. Thereafter, they were washed in consecutive steps serially with 10% SDS, 20‐mL 90% ethanol (for 45 s), 50‐mL sterile distilled water (washed thrice), 50 mL 2% (*v*/*v*) NaOCl (for 2 min), 50‐mL sterile distilled water (washed thrice), 50‐mL 70% ethanol (for 45 s), and finally five times with 50 mL of sterile distilled water. Germinated seedlings were placed in pots filled with a mixture of sterile sand and soil in the ratio of 3:1 (garden soil from the Jawaharlal Nehru University campus and acid washed riverbed sand). Plants were supplied with half‐strength modified Hoagland solution (5 mM KNO_3_, 5 mM Ca(NO)_2_, 2 Mm MgSO_4_, 10 M KH_2_PO_4_, 10 M MgCl_2_, 4 M ZnSO_4_, 1 M CaSO_4_, 1 M NaMoO_4_, and 50 M H_3_BO_3_) weekly. All the pots were grown for 40 days in a controlled environment chamber facility (CECF) maintained at daily temperature of 29^°^C ± 2^°^C. Plants were grown under controlled conditions in a greenhouse with an 8/16‐h light–dark photoperiod (light intensity of 1000 lux, using high‐intensity fluorescent light) at a temperature cycle of 25°C/18°C with a relative humidity 60%–70%. They were watered as needed.

### 4.6. Histochemical Analysis

Histochemical analysis of the roots of plants from all sets was done to check colonization. We selected 10 root samples randomly from each set; that is, two roots from each set were selected. Each set has three pots, and in each pot, five plants were grown. To study colonization, root sections of ~1 cm were placed in 2‐mL centrifugation tubes. They were incubated in 1‐mL 10% KOH solution at 60°C for 15 min to soften the plant material and thereafter neutralized in 1 N HCL for 5 min. The samples were then washed thrice with sterile distilled water and stained in 1‐mL 0.02% trypan blue at 60°C for 15 min. Samples were destained with 1‐mL 50% lactophenol for 15–60 min and mounted on a slide under a coverslip for observation with a light microscope. Percentage colonization for the inoculated plants was calculated using the following formula as described previously [8]:

%Colonization=root section with P.indicatotal root sections observed×100.

### 4.7. Determination of Biomass

Well‐grown chickpea plants from all the four sets were harvested for analyses after 40 days of growth. The pots were tapped lightly all across the outside to loosen the soil inside. A scraper was used to scrape the soil sticking on the inside walls. Each pot was held and gently turned upside down to remove the intact plant with its soil. The bulk soil around the roots was removed very carefully with hands so as to not damage the roots of the plant. The plants were washed gently multiple times to remove residual traces of soil sticking to the root. Washed plants were stretched lengthwise on blotting sheets to remove excess water. Plants from all the four sets were kept distinct on separate blotting sheets. Dry weight, number of branches, and shoot and root lengths of plants from each of these four sets were recorded. To measure the dry weight, harvested plants were washed with water and oven‐dried at 80°C for 72 h [10].

### 4.8. Statistical Analysis

GraphPad Prism was used to conduct the unpaired *t*‐test to check the significance.

## Funding

This study was supported by the University Grant Commission‐University Programs for Excellence‐II (UGC‐UPE‐II), Govt. of India, New Delhi, India, and Department of Science and Technology‐Promotion of University Research and Scientific Excellence (DST‐PURSE) Govt. of India.

## Disclosure

A preprint has previously been published [35].

## Conflicts of Interest

The authors declare no conflicts of interest.

## Data Availability

The data that support the findings of this study are available from the corresponding author upon reasonable request.
